# Investigation of the Infection of *Enterocytozoon bieneusi* in Sheep and Goats in Jiangsu, China

**DOI:** 10.3390/vetsci11070327

**Published:** 2024-07-19

**Authors:** Cheng Cheng, Yuan Cai, Hua Xing, Jianping Tao, Darong Cheng

**Affiliations:** 1 College of Veterinary Medicine, Yangzhou University, Yangzhou 225009, China; 13291399018@163.com (C.C.);; 2 Jiangsu Co-Innovation Center for Prevention and Control of Important Animal Infectious Diseases and Zoonoses, Yangzhou 225009, China

**Keywords:** *Enterocytozoon bieneusi*, sheep and goats, prevalence, genotype

## Abstract

**Simple Summary:**

*Enterocytozoon bieneusi* (*E. bieneusi*) is a parasite that infects both animals and humans. Understanding its distribution and genetic diversity is important for disease prevention and control. This study investigated the presence and genotypes of *E. bieneusi* in sheep and goats across five regions in Jiangsu Province, China. The research found that *E. bieneusi* was present in all tested regions, with infection rates varying from 23.65% to 42.81%. The overall infection rate was 36.51%. There was no significant difference in infection rates between sheep and goats or between animals of different ages, but health condition did affect infection rates. Six genotypes of *E. bieneusi* were identified, all belonging to a non-zoonotic group. These findings contribute to the better understanding and management of *E. bieneusi* infections in Jiangsu.

**Abstract:**

In order to investigate the infection status and genotypes of *Enterocytozoon bieneusi* (*E. bieneusi*) in sheep and goats in Jiangsu Province, a total of 786 fresh fecal samples from 18 farms across five regions in Jiangsu were collected and examined for the presence of *E. bieneusi*, and the genotype of *E. bieneusi* was examined using nested-PCR and sequencing of the ribosomal internal transcribed spacer. The results showed that *E. bieneusi* was detected in the fecal samples of sheep and goats in all regions, with infection rates ranging from 23.65% to 42.81%. The overall infection rate was 36.51% (287/786). The infection rate of *E. bieneusi* showed no significant difference between sheep and goats, as well as among different ages of animals (*p* > 0.05), but showed a significant difference in sheep and goats with different health conditions (*p* < 0.05). The positive products were amplified and cloned and subjected to sequenced analysis. Six genotypes, BEB6, CHG2, CHG3, CHC8, CHG14, and COS-I, were found. Phylogenetic analysis indicated that the six genotypes belonged to Group 2, which had previously been described as a non-zoonotic group.

## 1. Introduction

*Enterocytozoon bieneusi* is a zoonotic parasitic protozoan, infecting over 200 mammalian species including humans, domestic animals, and wild animals [1,2]. Hosts infected with *E. bieneusi* may experience acute and chronic diarrhea and dyspepsia, while immunocompromised hosts may suffer from fatal diarrhea. Thus, this parasite poses a significant threat to public health [3]. Due to its lack of staining characteristics and the small size of its spores, *E. bieneusi* is difficult to identify accurately under a microscope, making PCR detection a commonly used method for its identification. Based on the internal transcribed spacer (ITS) sequence of *E. bieneusi* rRNA, this parasite exhibits extensive genetic diversity, with at least 800 genotypes. Of these, 126 genotypes have been documented in humans, 614 in animals, and 58 in both humans and animals (indicating zoonotic potential) [4]. The genotypes D, EbpA, EbpC, Type IV, BEB6, O, J, CM4, Peru6, I, Peru8, Peru11, and BEB4 are the genotypes most commonly found in animals (including humans) [5]. Phylogenetic analysis has shown that the genotypes of *E. bieneusi* can be divided into nine different groups, and most genotypes in Group 1 can infect humans, posing a zoonotic risk, while groups 2–9 exhibit host specificity [6]. Recent studies have found that the host-specific subgroups BEB4, BEB6, and J genotypes can also infect humans [7,8]. In goats, 49 genotypes have been identified (44 found in China, with 12 being zoonotic), with BEB6 and CHG3 being the most common. In sheep, 79 genotypes have been documented (61 found in China, with 17 being zoonotic), with BEB6 and CM7 being the most common (Table S1) [4].

Spores are the infective stage of *E. bieneusi*. Human infections with *E. bieneusi* are often associated with contaminated water sources, and fecal–oral transmission is the main route of infection [9]. Immunocompetent individuals infected with *E. bieneusi* typically experience mild or self-limiting diarrhea, while immunocompromised individuals may develop more severe clinical symptoms such as vomiting, nausea, weight loss, dysenteric diarrhea, and fever. The spores dispersed in the environment have strong survival abilities, and there are currently no specific drugs for *E. bieneusi*, posing a significant health risk to farmers and workers. The sheep farming industry is an important part of modern animal husbandry. In recent years, with the adjustment and optimization of the agricultural industry structure and increasing consumer demand, sheep farming has developed rapidly [10]. With advancements in sheep farming practices and technology, the number of sheep and goats raised in Jiangsu Province has increased annually, reaching 6.306 million in 2022. *E. bieneusi* is a common pathogen in sheep. Mild infections can cause slow growth and development, while severe infections can lead to illness and even death [9,11,12]. This study aimed to investigate the infection status of gastrointestinal parasites in 18 goat and sheep farms across five cities in Jiangsu Province: Suzhou, Suqian, Nantong, Huaian, and Taizhou. The infection rate of *E. bieneusi* was determined and genotyped. This study provided supplementary data on *E. bieneusi* infection in Jiangsu Province. These data provide a theoretical basis for formulating effective prevention and control measures.

## 2. Materials and Methods

### 2.1. Chemicals and Samples Collection

**Figure 1:**
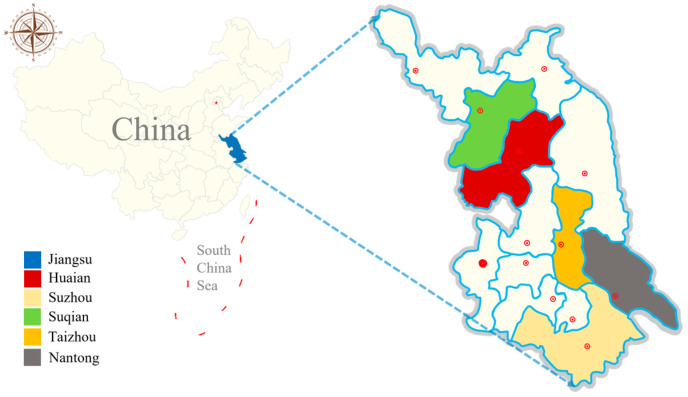
Distribution map of sampling districts of five cities in Jiangsu province, China.

Sample collection: In August 2022, a total of 786 fresh fecal samples were collected from 18 goat and sheep farms across five cities in Jiangsu Province: Suzhou, Suqian, Nantong, Huai’an, and Taizhou (Figure 1, Table 1). Some farms feed both sheep and goats, with sheep and goats being raised in different pens. Using the five-point sampling method for sampling, 50 g measures of fresh samples were taken from each pen (the animals in each pen were of the same month in age), placed in a clean sealed bag, and labeled with information including their collection location, age, and health condition (diarrheic pen or healthy pen: diarrheic pen: at least one sick animal and only collect diarrhea fecal samples; healthy pen: every animal is healthy) and a unique identifier.

Chemicals: EasyPure^®^ Quick Gel Extraction Kit (TransGen Biotech Co., Ltd., Beijing, China), DL2000 DNA Marker (Takara Bio Inc., Kusatsu, Shiga, Japan), DH5α Competent Cells (TransGen), Premix Taq™ (TaKaRa Taq™ Version 2.0) (Takara), pGEM-T easy Vector (Promega Biotech Co., Ltd., Beijing, China), and EasyPure^®^ Stool Genomic DNA Kit (TransGen).

### 2.2. Extraction of DNA and PCR Analysis

To extract DNA, around 200 mg of fecal sample was placed in a beaker and mixed with a small amount of sterile water. Then, 250 μL of this mixture was transferred to a 2 mL centrifuge tube, and DNA was extracted using the EasyPure^®^ Stool Genomic DNA Kit following the manufacturer’s guidelines. For the nested PCR amplification of the *E. bieneusi* ITS gene, primers were designed based on Buckholt’s methodology [13] (Table 2).

The first PCR amplification was performed in a 25.0 μL reaction mixture, containing 12.5 μL of Premix Taq, 1.0 μL of each primer (10 mmol/L), and 1.0 μL of template DNA and filled with double-distilled water to 25.0 μL. The cycling conditions were initial denaturation at 94 °C for 5 min, 35 cycles of 94 °C for 30 s, 57 °C for 30 s, 72 °C for 1 min, and a final extension at 72 °C for 10 min.

The second PCR amplification was performed in a 25.0 μL reaction mixture, containing 12.5 μL of Premix Taq, 1.0 μL of each primer (10 mmol/L), and 1.0 μL of template DNA (diluted 10-fold from the first round) and filled with double-distilled water to 25.0 μL. The cycling conditions were initial denaturation at 94 °C for 5 min, 35 cycles of 94 °C for 30 s, 55 °C for 30 s, 72 °C for 1 min, and a final extension at 72 °C for 10 min.

After PCR, 10 μL of the second-round product was run on a 1% agarose gel in TAE buffer at 120 V for about 30 min. The gel was visualized and photographed using a BIO-RAD UV imaging system. DL 2000 DNA Marker served as a reference, and positive bands were purified using the EasyPure^®^ Quick Gel Extraction Kit.

### 2.3. Phylogenetic Analysis and Statistical Analysis

The raw sequences obtained from sequencing were aligned using Clustal X (1.83) software. Homology comparisons of *E. bieneusi* ITS were performed against the GenBank database via Blast. Relevant reference sequences were retrieved to construct a phylogenetic tree for species identification using MEGA 7 (7.0.26) software.

Statistical analysis was performed with IBM SPSS Statistics 26 software, employing chi-square tests to examine potential factors influencing *E. bieneusi* infection rates in sheep and goats. This analysis helped us to identify the risk factors associated with *E. bieneusi* infection.

## 3. Results

### 3.1. PCR Amplification Results

Based on the ITS gene sequence of *E. bieneusi*, nested PCR detection revealed that 287 of the 786 fresh fecal samples collected were positive, with an overall infection rate of 36.51%.

### 3.2. Sequencing and Phylogenetic Tree Construction

**Figure 2:**
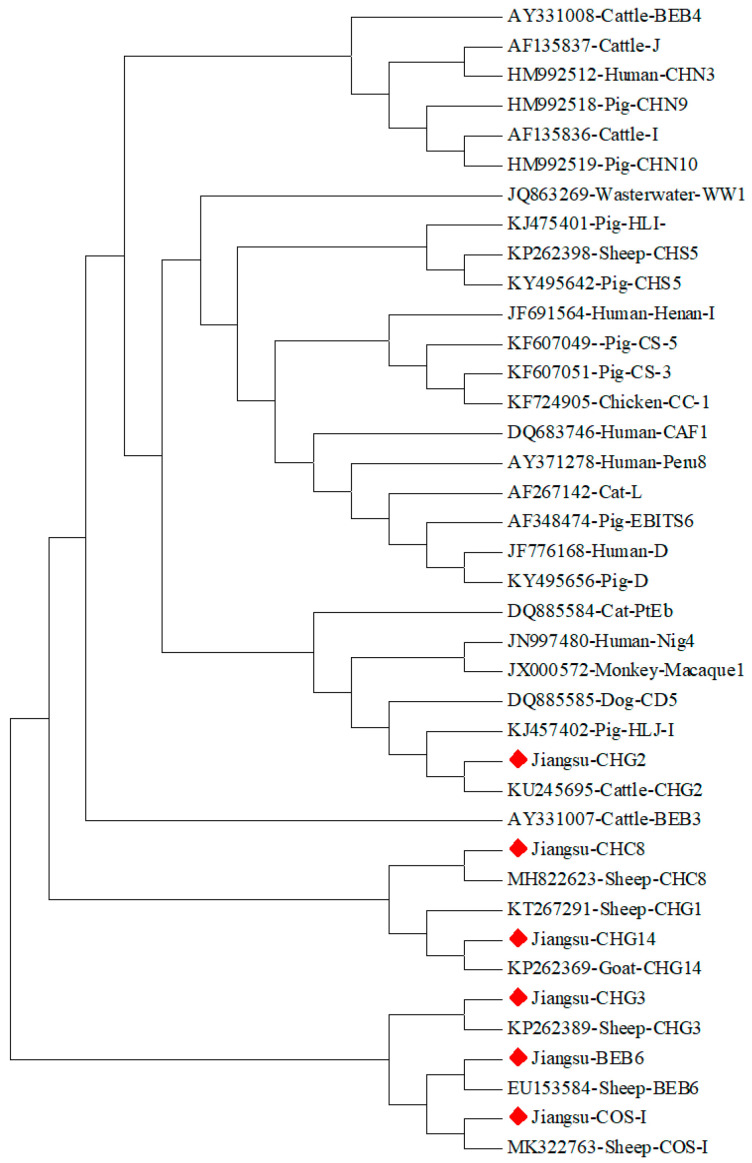
Phylogenetic tree of the ITS gene of the *E. bieneusi*, the red diamond is the sample in this study.

Sequence analysis of 96 *E. bieneusi* positive samples identified six genotypes through BLAST sequence comparison: BEB6, CHG2, CHG3, CHC8, CHG14, and COS-I. Phylogenetic tree construction using MEGA 7 showed that all six genotypes belonged to the host-specific evolutionary Group 2, with no genotypes being found in the other groups (Figure 2). Nantong had the most *E. bieneusi* genotypes (6), followed by Suzhou (4), Taizhou (3), Suqian (3), and Huaian (3). The comparison of *E. bieneusi* genotypes in sheep and goats showed that BEB6, CHG2, CHG3, CHC8, and COS-I were present in both species, while CHG14 was only detected in goats.

### 3.3. Infection Status of E. bieneusi in Sheep and Goats

Comparing the detection data from sheep farms in five different regions revealed differences in the infection rates of *E. bieneusi* in meat sheep (Table 3). The average infection rates in different regions ranged from 23.65% to 42.81%, with the highest being in Nantong at 42.81%, followed by Huai’an at 39.17%, Suzhou at 38.33%, Taizhou at 28.33%, and Suqian at 23.65%. The overall infection rates were similar between goats (35.54%) and sheep (38.89%), with no significant difference (*p* > 0.05) (Table 4). In goats, higher infection rates were observed in those aged 0–2 months (37.39%) and 2–6 months (39.26%) compared to those aged 6–10 months (25%). In sheep, the highest infection rate was in the 2–6-months-old age group (75%), followed by those aged 0–2 months (40%) and 6–10 months (34.18%). However, there was no significant difference in infection rates among different age groups in both sheep and goats (*p* > 0.05) (Table 5). When considering health conditions, diarrheic goats showed a higher infection rate than healthy goats, whereas in sheep, healthy sheep had a higher infection rate compared to diarrhea sheep. This variation in infection rates based on health conditions was significant (*p* < 0.05) (Table 6).

## 4. Discussion

*E. bieneusi* is a zoonotic parasite that inhabits the intestines of humans and various animals [14]. Currently, reports of *E. bieneusi* in sheep and goats are increasing worldwide, with most cases in sheep and goats in China being reported in the central, eastern, and southwestern regions [15,16,17,18,19,20,21]. This study shows that the overall infection rate of *E. bieneusi* in sheep and goats in Jiangsu is 36.51% (287/786), higher than the rates reported in 2019 for Xuzhou, Jiangsu (2.7%), and Anhui (4.09%) [7] but lower than the infection rates in Henan (73.6%), Chongqing (62.5%), and Shanxi (47.8%, 43.5%) [15,16]. The infection rates of *E. bieneusi* vary by region, with the highest rate found in Nantong (42.81%) and the lowest found in Suqian (23.65%). There are also significant differences in infection rates among farms within the same region. For example, the infection rate at Farm 14 in Suqian (37.5%) is four times that of Farm 13 (10.42%), and the infection rate at Farm 11 in Taizhou (53.33%) is nearly 20 times that of Farm 12. The large differences in infection rates may be related to the management practices and breeding methods of the farms, such as overcrowding, failure to ensure timely cleaning of the fecal waste in pens, and contamination of feed. In this study, the infection rate of *E. bieneusi* was higher in lambs aged 0–2 months old and fattening sheep aged 6–10 months old compared to growing sheep aged 2–6 months old. Zhang et al. discovered in their study of Tibetan sheep and yaks in Tibet that the infection rate of *E. bieneusi* was highest in animals under 1 year old, followed by those aged 1–2 years old, and lowest in those over 2 years old [6]. Fiuza et al. found that in their survey of sheep on 10 farms in Brazil, the infection rate in lambs (less than 6 months of age) (34.1%) was higher than that in older sheep (11.1%) [22]. Survey results of samples collected from various regions in China indicate that the infection rate decreases with age [23]. Analysis suggests that lambs under 10 months old are particularly susceptible to *E. bieneusi*, possibly due to a decrease in maternal antibodies, making them more prone to infection. Therefore, we should enhance the feeding management of sheep under 10 months old, provide adequate nutrition, and raise them in separate pens to reduce the risk of parasitic infections in young animals. Additionally, the infection rate in goats with diarrhea was higher than in healthy goats, while in sheep, the infection rate was lower in those with diarrhea compared to healthy ones, suggesting that diarrhea may result from other intestinal pathogens, such as *Eimeria* spp., *Trichostrongylidae* spp., etc. In addition, the small size of diarrhea samples from sheep may have produced false-negative results, making it difficult to extract patterns from them, which may also explain the lower positive rate of diarrhea samples compared to healthy samples. Therefore, in future study, it will be necessary to increase the sample size collected from sheep and goats with different symptoms and test other pathogens in sheep and goats.

Currently, 79 *E. bieneusi* genotypes have been identified in sheep, and 49 genotypes have been identified in goats. Of these, 15 genotypes are shared between sheep and goats, namely BEB6, CHC8, CHG1, CHG13, CHG2, CHG3, CHS5, CHS7, CM7, COS-I, COS-II, D, EbpA, EbpC, and Peru6. Wu et al. tested 177 samples from Tibetan sheep in Gansu, identifying four genotypes, namely BEB6, CM7, CHS3, and CGS1, all of which belong to Group 2, with BEB6 being the dominant genotype [24]. Zhao et al. investigated 193 fecal samples from eight farms in Heilongjiang, identifying 14 genotypes, with 6 known (BEB6, Peru6(8), D, O, EbpC, EbpA) and 8 novel genotypes (COS-I to COS-VII and COG-I) [25]. Xie et al. collected 907 fecal samples from black goats in five counties in Yunnan Province, identifying 15 genotypes of *E. bieneusi*: 4 novel (CYG-1, CYG-2, CYG-3, CYG-4) and 11 known (CHG1, CHG2, CHG3, CHG5, CHG28, J, D, BEB6, Wildboar3, CD6, SDD1) genotypes [21]. Yang et al. tested sheep fecal samples from 11 provinces in China and found that all regions with positive samples had the BEB6 genotype as the dominant genotype [26]. Our study identified six genotypes in 287 positive samples, including BEB6, CHG2, CHG3, CHC8, CHG14, and COS-I. BEB6 was the most dominant genotype (55.21%), followed by CHG3 (25%). BEB6, CHG2, CHG3, CHC8, and COS-I were detected in both sheep and goats, while CHG14 was only found in goats. Phylogenetic analysis showed that all identified genotypes belong to the Group 2 subgroup, which is not zoonotic, but BEB6 poses a zoonotic risk. Additionally, in China, BEB6 has been reported in other animals such as Bactrian camels, red deer, Siberian roe deer, sika deer, and Altai marmots, posing a significant threat to public health [27,28,29,30]. Therefore, enhancing the monitoring of *E. bieneusi* and regularly providing epidemiological data to track regional dynamics is crucial for public health.

## 5. Conclusions

The study on *E. bieneusi* infection in sheep and goats in Jiangsu revealed an overall infection rate of 36.51%. Six genotypes of *E. bieneusi* were identified, with BEB6 being the dominant genotype and posing a potential zoonotic risk. Improving the management of sheep and goats under 10 months of age and enhancing the monitoring of other pathogens is necessary. The findings underscore the importance of monitoring *E. bieneusi* as a zoonotic pathogen. Regular epidemiological surveys and data collection are crucial for tracking regional dynamics and protecting public health.

## Figures and Tables

**Table 1:** Sample information.

Region	0~2 Month	2~6 Month	6~10 Month	Breed
Suzhou	90	90	0	Goats
Nantong	80	80	118	Sheep and goats
Taizhou	0	0	60	Sheep
Suqian	20	40	88	Sheep and goats
Huaian	60	0	60	Sheep and goats
Total	250	210	326	

**Table 2:** Primers for nested-PCR amplification of *E. bieneusi*.

Gene Locus	Primer	Sequence	Amplified Product
ITS	NEBF1	5′-GGTCATAGGGATGAAGAG-3	410 bp
	NEBR1	5′-TTCGAGTTCTTTCGCGCTC-3′	
	NEBF2	5′-GCTCTGAATATCTATGGCT-3′	392 bp
	NEBR2	5′-ATCGCCGACGGATCCAAGTG-3′	

**Table 3:** Infection of *E. bieneusi* in mutton sheep in different areas.

Region	Farm	Sample Size	No of Positive	% of Positive	Average Positive %
Suzhou	1	60	20	33.33	38.33
	2	60	19	31.67	
	3	60	30	50	
Nantong	4	80	24	30	42.81
	5	60	17	28.33	
	6	37	8	21.62	
	7	30	20	66.67	
	8	21	12	57.14	
	9	30	23	76.77	
	10	20	15	75	
Taizhou	11	30	16	53.33	28.33
	12	30	1	3.33	
Suqian	13	48	5	10.42	23.65
	14	40	15	37.5	
	15	60	15	25	
Huaian	16	40	26	65	39.17
	17	40	17	42.5	
	18	40	4	10	
Total		786	287	36.51	

**Table 4:** Infection of *E. bieneusi* in sheep and goats.

Breed	Sample Size	No of Positive	% of Positive
Goat	588	209	35.54
Sheep	198	77	38.89
total	786	287	36.51

**Table 5:** Infection of *E. bieneusi* in mutton sheep of different months in age.

Breed	Age (Month)	Sample Size	No of Positive	% of Positive
Goat	0–2	230	86	37.39
	2–6	242	95	39.26
	6–10	116	29	25
Sheep	0–2	20	8	40
	2–6	20	15	75
	6–10	158	54	34.18

**Table 6:** Correlation of *E. bieneusi* infection and diarrhea in animals.

Breed	Symptom	Sample Size	No of Positive	% of Positive
Goat	Diarrheic	140	62	44.29
	Healthy	448	148	33.04
Sheep	Diarrheic	38	8	21.05
	Healthy	160	69	43.13

## Data Availability

The raw data supporting the conclusions of this article will be made available by the authors, without undue reservation.

## Supplementary Materials

The following supporting information can be downloaded at: https://www.mdpi.com/article/10.3390/vetsci11070327/s1, Table S1: Genotypes of *E. bieneusi* infection in human, goat, and sheep.
